# Study of hub nodes of transcription factor-target gene regulatory network and immune mechanism for type 2 diabetes based on chip analysis of GEO database

**DOI:** 10.3389/fmolb.2024.1410004

**Published:** 2024-05-24

**Authors:** Guangyu Xu, Yuehan Zhao, Yu Bai, Yan Lin

**Affiliations:** ^1^ College of Pharmacy, Beihua University, Jilin, China; ^2^ College of Pharmacy, Jilin Medical University, Jilin, China; ^3^ School of Basic Medical Sciences, Beihua University, Jilin, China

**Keywords:** type 2 diabetes, GEO database, transcription factors, network regulation, immune mechanism

## Abstract

Identification of novel therapeutic targets for type 2 diabetes is a key area of contemporary research. In this study, we screened differentially expressed genes in type 2 diabetes through the GEO database and sought to identify the key virulence factors for type 2 diabetes through a transcription factor regulatory network. Our findings may help identify new therapeutic targets for type 2 diabetes. Data pertaining to the humoral (whole blood) gene expression profile of diabetic patients were obtained from the NCBI’s GEO Datasets database and gene sets with differential expression were identified. Subsequently, the TRED transcriptional regulatory element database was integrated to build a gene regulatory network for type 2 diabetes. Functional analysis (GO-Analysis) and Pathway-analysis of differentially expressed genes were performed using the DAVID database and the Kyoto Encyclopedia of Genes and Genomes (KEGG) database. Finally, gene-disease correlation analysis was performed using the DAVID online annotation tool. A total of 236 pathogenic genes, four transcription factors related to the pathogenic genes, and 261 corresponding target genes were identified. A transcription factor-target gene regulatory network for type 2 diabetes was constructed. Most of the key factors of the transcription factor-target gene regulatory network for type 2 diabetes were found closely related to the immune metabolic system and the functions of cell proliferation and transformation.

## 1 Introduction

Globally, an estimated 366 million people are affected by diabetes mellitus and its incidence has shown an increasing trend (Joanne and Jose, 2020). Diabetes ranks as the third most harmful disease after cancer and coronary atherosclerotic heart disease (Chen et al., 2017). The high incidence, high disability rate, and lifelong harm caused by diabetes imposes a heavy economic burden on the society and families (Le et al., 2018). Type 2 diabetes accounts for over 90% of all diabetic patients (Shen et al., 2018). Therefore, the prevention and treatment of type 2 diabetes have attracted great attention from domestic and overseas scholars and governments (Wang and Wei, 2017).

Type 2 diabetes is a complex metabolic disorder involving the metabolism of sugars, proteins, fat, water, and electrolytes. The condition results from hypofunction of the pancreas and insulin resistance; the underlying etiopathogenetic mechanisms are complex and involve genetic factors, immune disorders, infection, and toxins (Guess, 2018). Traditional single gene screening for type 2 diabetes has been unable to meet the needs of clinical medicine (Yi et al., 2022; Kaul and Ali, 2015).

Homeostasis regulation depends on common pathways of the metabolic and immune systems, and metabolic regulation and immune response interact. When dysfunction occurs, it can lead to chronic metabolic disorders in the body. If endogenous or exogenous infections can cause immune responses and metabolic disorders, metabolic abnormalities that occur when nutrient and energy intake and expenditure are out of balance can also induce immune responses.

Transcription factors are a class of proteins with DNA-binding domains that bind to specific DNA sequences and regulate gene transcription by promoting or preventing the recruitment of RNA polymerase. Transcription factors play an important regulatory role in complex networks through thousands of genomic binding sites. Therefore, the construction of a regulatory network of transcription factors may facilitate the identification of novel diagnostic and therapeutic targets for type 2 diabetes (Da et al., 2017).

In this study, the humoral (whole blood) gene expression profile of type 2 diabetic patients was obtained through the GEO Datasets database of the National Center for Biotechnology Information (NCBI), and the differentially expressed gene sets were selected. Subsequently, the TRED transcriptional regulatory element database was integrated to build a gene regulatory network for type 2 diabetes based on the differentially expressed gene set. Gene-disease correlation analysis was performed using the DAVID online annotation tool. Our findings may help identify some novel diagnostic targets and lay the foundation for early clinical diagnosis of type 2 diabetes and the development of novel drugs.

### 1.1 Methodology

#### 1.1.1 Microarray data

The microarray data used in this study was obtained from GEO Datasets of the NCBI database (Barrett et al., 2013); the index word used was “Diabetic”. The filter subjects were “*Homo sapiens*,” “CEL original document,” and “Affymetrix”. Three groups of microarrays were eventually identified.

We chose gene expression profiles of GSE15653, GSE64998, and GSE23343 from the GEO database, which are freely available in the public domain. The GSE15653 datasets were based on the Affymetrix GPL96 platform and included 13 samples (4 diabetic samples and nine healthy samples). The GSE64998 datasets were based on the Affymetrix GPL11532 platform and included 13 samples (7 diabetic samples and six healthy samples). The GSE23343 datasets were based on the Affymetrix GPL570 platform and included 17 samples (10 diabetic samples and seven healthy samples) (Table 1).

**TABLE 1:** Microarray data.

Dataset ID	Sample ID	Sample number	Control sample number	Disease sample number	Platforms	Organism	Submission date	Manufacturer
GSE64998	GSM1585585-GSM1585597	13	6	7	GPL11532	*Homo sapiens*	08 Mar 2016	Affymetrix
GSE15653	GSM391698-GSM391710	13	9	4	GPL96	*Homo sapiens*	01 Jun 2009	Affymetrix
GSE23343	GSM572800- GSM572816	17	7	10	GPL570	*Homo sapiens*	31 July 2010	Affymetrix

#### 1.1.2 Microarray data processing

The Expression Console™ software tool of Affymetrix was used to perform background correction and probe fluorescence conversion to microarray data. The Transcriptome Analysis Console tool of Affymetrix was used to standardize and perform logarithmic conversion of the microarray data. The significance analysis of microarrays (SAM) method was used to identify differentially expressed mRNA between healthy individuals and patients with type 2 diabetes. Fold change >1.0 or fold change < −1.0 and *p*-value <0.05 were used as the criteria to identify differentially expressed genes in this study.

#### 1.1.3 Construction of TF mRNA gene network

Based on mRNA expression profiles after microarray data analysis and after searching the Transcriptional Regulatory Element Database (TRED) (Zhao et al., 2005), we obtained four transcription factors (TFs) and 236 target genes. Four transcription factors (TF) and 236 target genes were predicted to combine for a total of 261 TF-to-target pairs. The relation obtained from the analysis of the differential co-expression was mapped to the transcription factors and target gene pairs to obtain transcription regulation pairs. Finally, Cytoscape3.9.1 software (Shannon et al., 2003) was used for plotting. The yellow rhombus in the TF-gene network represented transcription factors and the blue rhombus represented target genes. The TFs as well as their target genes were connected by dotted lines with arrows indicating the direction from the source to the target.

#### 1.1.4 Construction of PPI network

The genes with more than 15 nodes were input into the String database (https://stringdb.org/) to construct the PPI network, the species was selected as *H. sapiens*, the score was set to ≥0.9, and other parameters were set as default.

#### 1.1.5 Gene function annotation analysis

The DAVID (Database for Annotation Visualization and Integrated Discovery) database (Huang et al., 2007) was used for the Gene Ontology (GO) function annotation enrichment analysis and Kyoto Encyclopedia of Genes and Genomes (KEGG) pathway analysis (Xu et al., 2014) on the screened differential genes. According to the GO significance reflected by the differentially expressed genes (*p* < 0.05), the differentially expressed genes were further analyzed from the functional perspective.

## 2 Results

### 2.1 Microarray data processing

**FIGURE 1:**
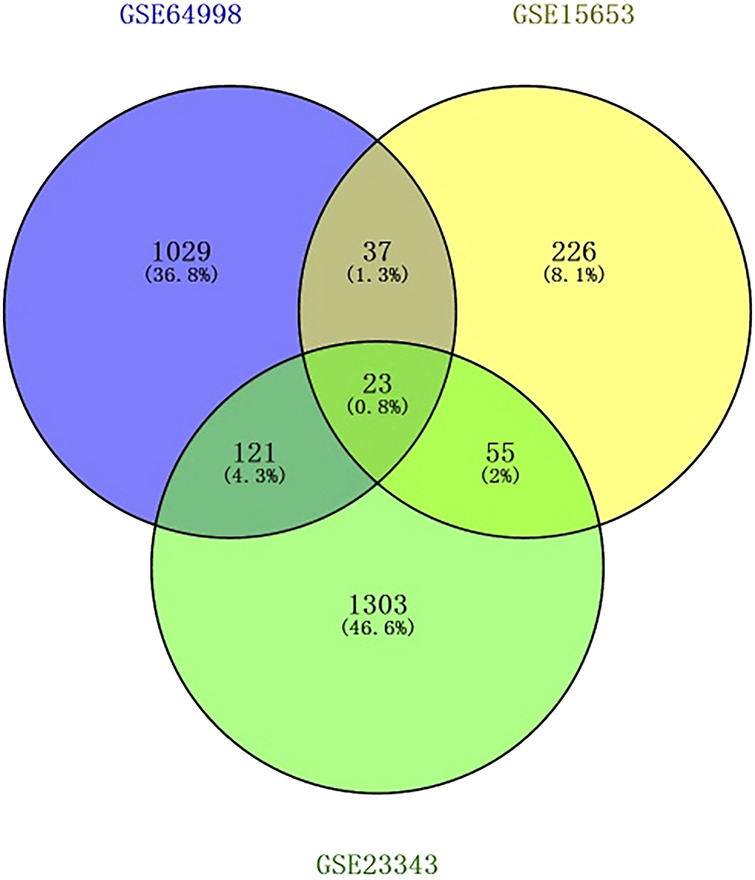
Screening results of differentially expressed genes overlapping on two or more platforms. The blue color is GSE15653, the yellow color is GSE23343, and the green color is GSE64998.

**FIGURE 2:**
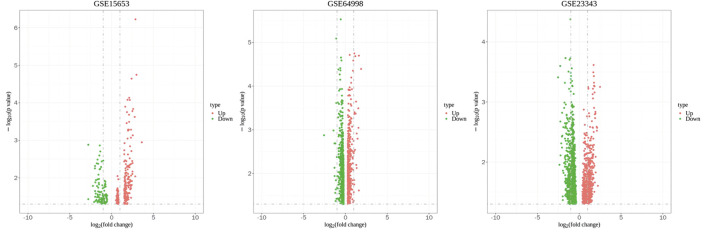
Volcano plot of GSE15653, GSE64998 and GSE23343. The red represents upregulated differentially expressed genes, and the green represents downregulated differentially expressed genes.

The Transcriptome Analysis Console tool of Affymetrix was used to standardize and perform logarithm conversion of the microarray data, while the SAM method was used to identify the differentially expressed miRNAs between healthy individuals and patients with type 2 diabetes. The numbers of differentially expressed genes on each platform were 1,210, 341, and 1,502, respectively. These genes were cross-screened and a total of 236 genes were identified (Figures 1, 2). Of these, 23 differentially expressed genes were identified on three platforms, while 37, 121, and 55 differentially expressed genes were identified on two platforms.

### 2.2 Transcription factor-target gene regulatory network for type 2 diabetes

The TRDE database was used to predict the possible transcription factors for the 236 genes; a total of four transcription factors and 261 corresponding target genes were identified (Table 2).

**TABLE 2:** Four transcription factors and their corresponding target genes.

TF	Target gene no.	Gene ID	Description
Jun	78	16,476	jun proto-oncogene
Stat1	66	20,846	signal transducer and activator of transcription
Fos	69	14,281	FBJ osteosarcoma oncogene
Atf5	48	107,503	sactivating transcription factor 5

**FIGURE 3:**
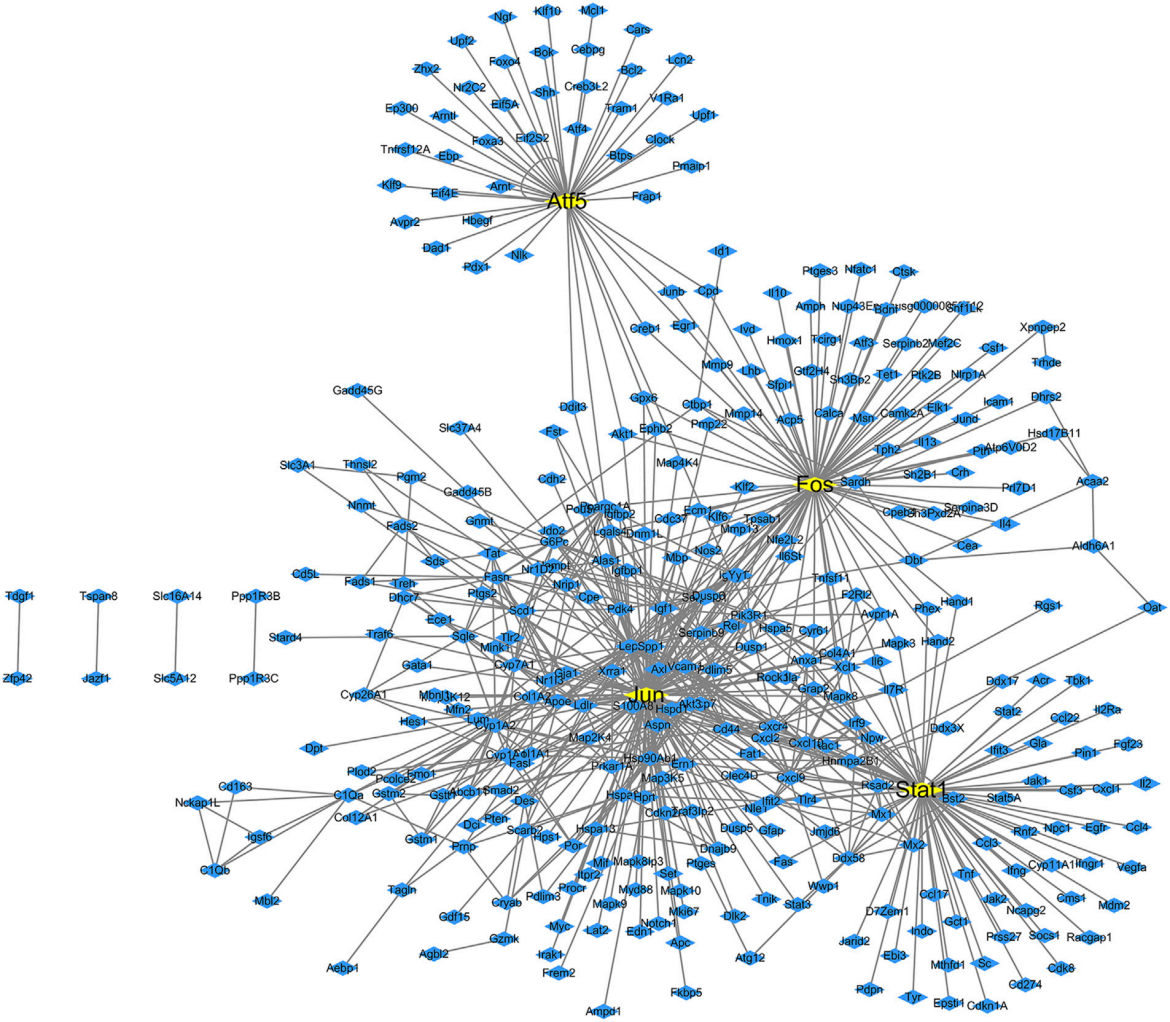
Transcription factor regulatory network map of differentially expressed genes in diabetes. The yellow boxes are transcription factors, while the blue boxes are targets genes.

The transcription factors and their corresponding 261 target genes were mapped using the cytoscape software (Figure 3). In Figure 3, the number of target genes regulated by transcription factor Jun is the largest, followed by Stat1, Fos and Atf5 (Table2).

We found that 13 target genes were regulated by more than two transcription factors (Table 3); of these, Pik3r1 (regulated by four transcription factors) was the most regulated target gene in this network. There were eight target genes regulated by three transcription factors and four target genes regulated by two transcription factors. The transcription factor Fos gene has a regulatory effect on all target genes.

**TABLE 3:** Target genes regulated by transcription factors.

Target genes regulated by transcription factors	Number of transcription factors that regulate target genes	Transcription factors	Number of network nodes
Pik3r1	4	Atf5 Jun Fos Stat1	22
Ephb2	3	Fos Jun Atf5	3
Gpx6	3	Fos Jun Atf5	3
Rela	3	Jun Fos Stat1	3
Mapk8	3	Jun Fos Stat1	3
Il6	3	Jun Fos Stat1	3
Mapk3	3	Jun Fos Stat1	3
Col4a1	3	Jun Fos Stat1	3
Grap2	3	Jun Fos Stat1	3
Junb	2	Fos Atf5	2
Creb1	2	Fos Atf5	2
Cpd	2	Fos Atf5	2
Egr1	2	Fos Atf5	2

### 2.3 Node of network

We performed a statistical analysis of the network nodes in the transcription factor-target gene regulatory network for type 2 diabetes. We identified 14 genes that existed in more than 15 nodes and three (Jun, Fos and Stat1) of these existed in more than 80 nodes; among these, Jun had the most network nodes (115 network nodes) (Table 4).

**TABLE 4:** Statistical table of transcription factor network nodes (>15 nodes).

Gene	Nodes of network	The number of regulatory transcription factors
Jun	115	1
Fos	88	1
Stat1	83	1
Atf5	48	1
Lepr	43	3
Hsp90ab1	32	3
Igf1	23	3
Pik3r1	22	4
Cxcl10	19	2
Cyp7a1	17	1
Serpine1	16	2
Apoe	15	1
Cyp1a2	15	1
Fasn	15	0

Furthermore, we found that Lepr, Hsp90ab1, Igf1 and Pik3r1 were closely related to the regulation of multiple transcription factors.

### 2.4 Construction of PPI network

**FIGURE 4:**
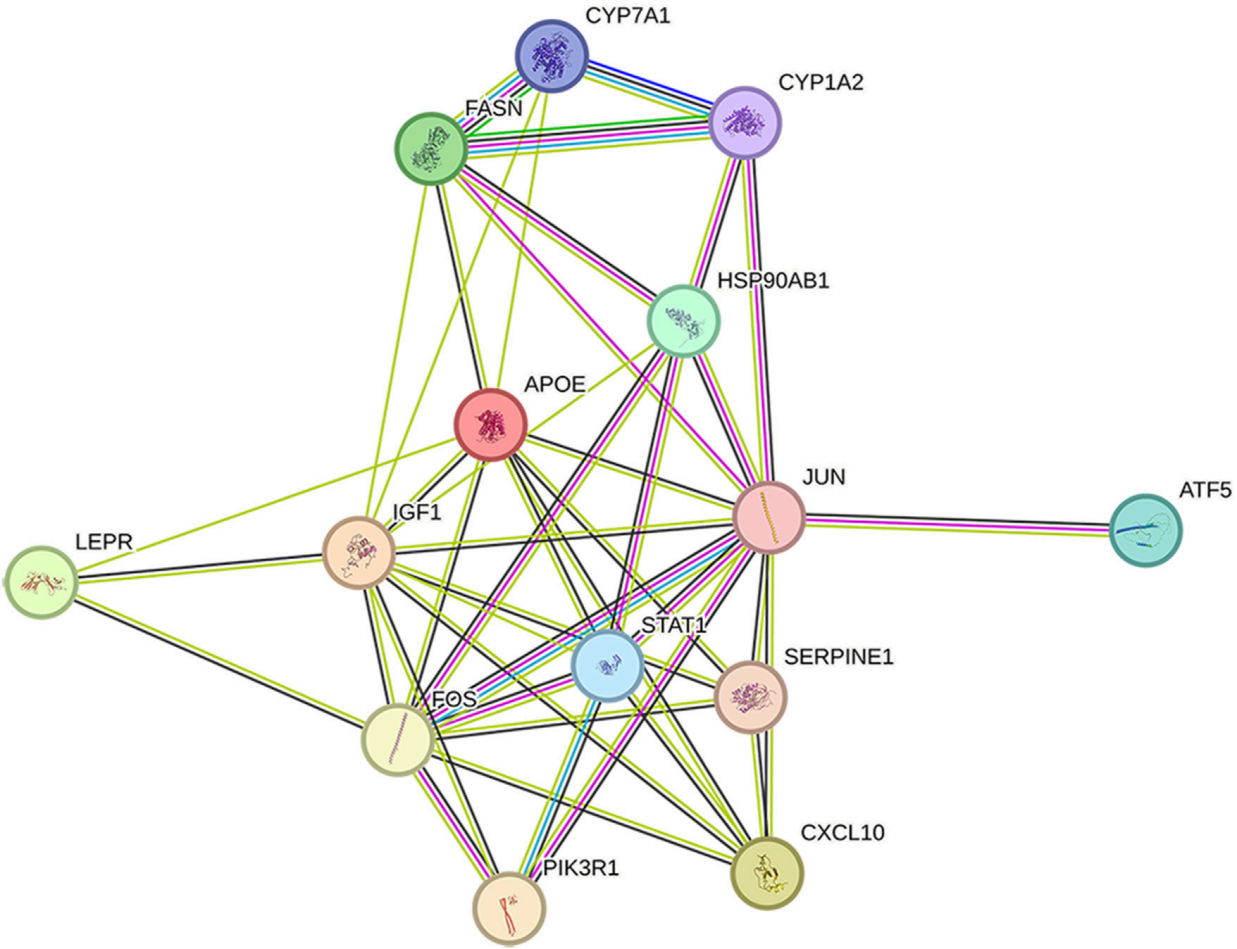
PPI network diagram.

The 14 key nodes (Table 4) were input into the String database to construct the PPI network (Figure 4), and it was found that JUN, STAT1, FOS, PIK3R1, ATF5 were closely related in the PPI network, and they were regarded as the key targets of type 2 diabetes.

### 2.5 GO functional annotation analysis of differentially expressed genes in diabetes

**FIGURE 5:**
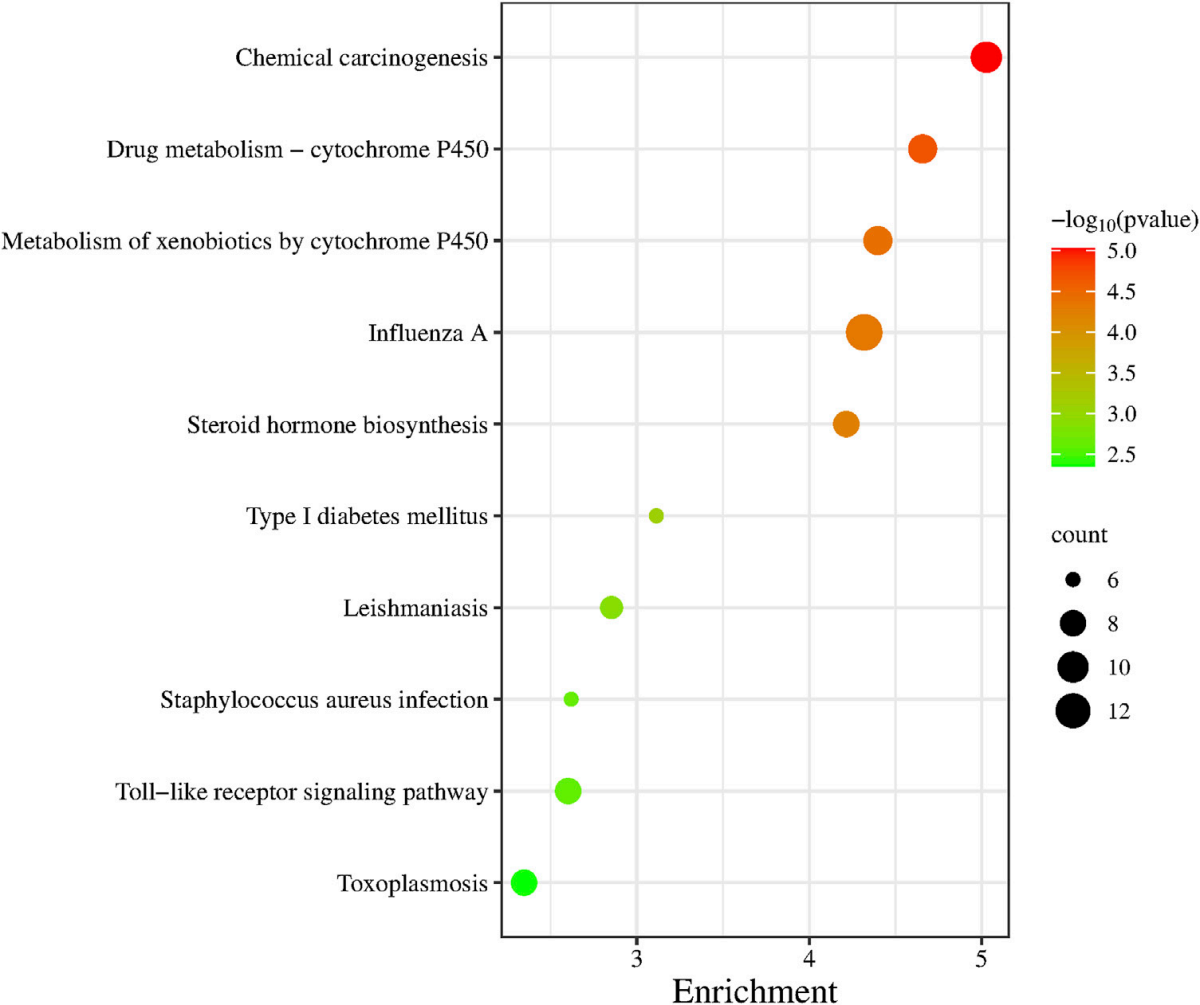
KEGG pathway enrichment analysis.

GO functional annotation was performed on 236 differentially expressed genes and the first 10 pathways were sequenced according to the *p*-value (Figure 5). Of those 10 pathways, Drug Metabolism-Cytochrome P450 pathway, Metabolism of xenobiotics by cytochrome P450 pathway, Steroid hormone biosynthesis pathway and Type I These four pathways are closely related to the metabolic system. The Influenza A pathway, *Staphylococcus aureus* infection pathway, Toxoplasmosis pathway and Leishmaniasis pathway are all pathogenic infectious diseases that are closely related to the body’s immune system.

## 3 Discussion

The prevalence of diabetes has shown a rapid increase owing to sedentary lifestyles in modern society and progressive population aging. Development of new hypoglycemic drugs and the identification of novel drug targets are a key areas of contemporary research (Vyas et al., 2015). With the advent of the post-genome era and the rapid development of bioinformatics, it is possible to construct information networks based on big data and to identify potential drug targets through network nodes (Li, 2012).

In this study, microarray data pertaining to type 2 diabetes was obtained from the GEO Datasets of the NCBI database. Further, The Transcriptome Analysis Console tool of Affymetrix was used to standardize and logarithmize the microarray data. A total of 236 differentially expressed pathogenic genes for diabetes were identified. These 236 genes were analyzed using the TRDE database, and four transcription factors (Jun, Stat1, Fos and Atf5) and their 261 corresponding target genes were predicted. Lastly, a transcription factor-target gene regulatory network for type 2 diabetes was constructed (Table 2).

Jun is closely related to systemic lupus erythematosus (SLE). SLE is a typical autoimmune disease involving multiple organs and systems. Doníz-Padilla et al. found that the expression level of Jun in peripheral blood mononuclear cells (PBMC) of patients with SLE was significantly higher than that in normal controls (Doniz et al., 2011; Linan et al., 2015). Olferiev et al. showed that Jun may play an important role in transcriptional regulation of FCGR2B promoter activity; FCGR2B has been shown to be closely related to the pathogenesis of SLE (Olferiev et al., 2007). These studies suggested that jun may be involved in the pathogenesis of SLE.

Gene Stat1 translated as STAT1, is a transport protein for interferon (Dale et al., 2015; Halupa et al., 2005) subsequent research showed that it is an important component of cellular response to interferon stimulation. STAT1 belongs to the STAT transcription factor family, which includes STAT1, STAT2, STAT3, STAT4, STAT5α, STAT5β, and STAT6. STAT1 plays a key role in cellular immune response against viruses, bacteria, and parasites (Chauche et al., 2017).

As a member of the Fos family, Fos along with the members of the Jun family and the activated transcription factor protein family were shown to form activated protein 1 (ap-1) (Wang et al., 2006). Activator protein-1 (ap-1) is an important intranuclear transcription regulator that plays an important role in many signal transduction processes; it represents the intranuclear intersection of a series of cell signal transduction pathways.

Gene Atf5 translated as ATF5 (activating transcription factor 5), is a member of the ATF/CREB (camp response element binding protein) family. In previous studies, full-length TRB3 was used as the decoy protein to screen the human liver cDNA gene bank and to identify the interaction between TRB3 and ATF5 (Yasuda et al., 2014). However, no studies have specifically reported the interaction between the two. Therefore, we concluded that the ATF5 protein has an unpredictable role in glucose metabolism or lipid metabolism. In particular, the function of ATF5 in preadipocyte differentiation through its interaction with TRB3 has not been reported.

After statistical analysis of the target genes regulated by transcription factors, we found that only one target gene (Pik3r1) was regulated by the four transcription factors, while eight target genes were regulated by three transcription factors. Pik3r1 is regulated by all four transcription factors and has 22 network nodes; therefore, it seems to play an important role in the transcription factor regulatory network. Pik3r1 is a member of the PI3K family, which is an important kinase of inositol and fluidomyositol (PI). As an important intracellular signal transduction molecule, Pik3r1 is involved in the processes of cell proliferation, apoptosis, and differentiation (Zhang et al., 2019). An increasing body of evidence suggests that Pik3r1 plays an important role in tumor biomolecular mechanisms (Vander et al., 2015). Several studies have shown that diabetes, particularly type 2 diabetes, is associated with an increased risk of breast, colorectal, endometrial, pancreatic, liver, and gallbladder cancer (Sanae et al., 2019; Zhao et al., 2021).

We also performed statistical analysis pertaining to the nodes of the ranscription factor-target gene regulatory network for type 2 diabetes; we found 14 genes that existed in more than 15 nodes, while eight genes existed in more than 20 nodes, (Jun, Fos, Stat1, Atf5, Lepr, Hsp90ab1, Igf1, and Pik3r1). The greater the number of nodes, the more important is the gene in the regulatory network. Of the eight genes with the largest number of nodes in the network, the first four were transcription factors (as discussed earlier) that were closely related to the immune system and signal transduction. Of the remaining four genes, Lepr plays an important role in maintaining energy hemostasis in the body. Ridker et al. identified the expression of LEPR in pancreatic β cells and found that it regulates insulin secretion in consort with leptin (Ridker et al., 2008). In addition, animal experiments have shown that the variation of LEPR plays an important role in the pathogenesis of obesity and diabetes in mice (Brito et al., 2016). Heat shock protein 90 (Hsp90) is widely found in eukaryotic and prokaryotic cells and is the most active molecular chaperone in the cytoplasm. Human Hsp90 is divided into two categories, Hsp90AA1 and Hsp90AB1, based on whether it contains abundant glutamine fragments. The Hsp90AB1 gene has been implicated in the pathogenesis of SLE *via* regulating the expression of Hsp90 through translation, which increases the expression of Hsp90 and interleukin-6 (IL6); this induces the differentiation of B lymphocytes into plasmocytes, promotes the production of autoantibodies, reduces the activity of CD8^+^ inhibitory T cells, and increases the secretion of immunoglobulins (Stephanou et al., 1998). As translated as insulin-like growth factor 1 (Igf1), IGF1 can promote cell proliferation and inhibit apoptosis; in addition, its role in tumor development is a hot topic in contemporary research. The biological function of IGF1 is mediated by its surface specific target cell receptor (IGF1R), which plays an important role in cell transformation and tumorigenesis in many tissues including ovaries; in addition, it can activate the MAPK and PI3K/AKT signaling pathways (Cheng et al., 2022). AKT is protein kinase B, which regulates cell proliferation, apoptosis, and cell cycle (Jae et al., 2020). We have also discussed that Pik3r1 gene is involved in regulating a variety of cellular functions including cell growth, proliferation, transformation and survival; it also plays an important role in tumor biomolecular mechanisms. To summarize, these eight genes are mainly involved in the immune response, cell proliferation, transformation, and other functions, all of which are closely related to type 2 diabetes.

We performed gene ontology (GO) functional annotation analysis of 236 differentially expressed genes, and the first 10 pathways were obtained according to the *p* values (Figure 5). These 10 pathways were closely related to diabetes and related diseases, of which the type 1 diabetes pathway is the sixth, and the toll-like receptor signaling pathway is closely related to immunity.

**FIGURE 6:**
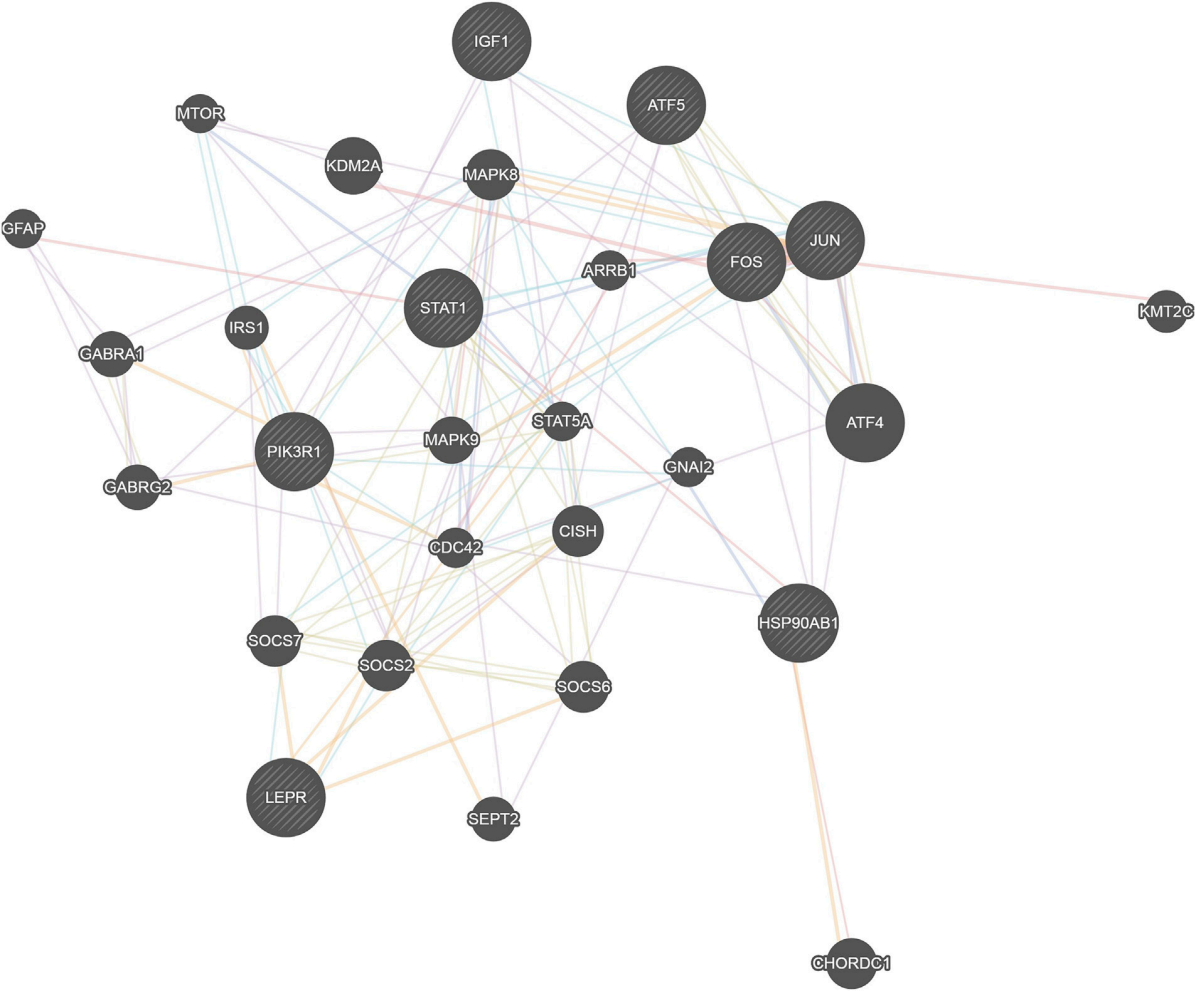
Network map of the relationship between transcription factors and network nodes. The circles represent transcription factors; the size of the circle is directly proportional to the number of genes it is linked with.

Eight network nodes including four transcription factors (Jun, Stat1, Fos and Atf5) that regulate the most target genes, the target gene that was most regulated by transcription factors (Pik3r1), and the eight genes with the most network nodes (Jun, Fos, Stat1, Atf5, Lepr, Hsp90ab1, Igf1, and Pik3r1) were put together for network construction analysis (Figure 6). We found that these genes were closely related to and were regulated by Stat5a, Mapk9 and Mapk8, all of which play a role in the STAT and MAPK signaling pathways for regulation of immune and inflammatory-related functions. At the same time, we also found that the two genes Stat1 and Pik3r1 were located in the central location of the networks, which indicated their importance.

Type 2 diabetes is generally believed to be related to the abnormal cell structure caused by the interaction of inflammatory factors with the endocrine system, immune system, oxidative stress, abnormal fat metabolism, and other factors; in addition, insulin resistance and microvascular disease plays a key role in its pathogenesis (Hiroki and Kazuhiro, 2022). We performed GO functional enrichment analysis of 236 differentially expressed genes through the DAVID website and selected the top 10 pathways associated with the most significant *p*-value. We found that most of these 10 pathways were related to immune, metabolism, and diabetes (such as the toll-like receptor signaling pathway and the type I diabetes mellitus pathway). There were also some transcription factors and signaling pathways related to diseases and immunity, such as Drug kibone-cytochrome P450, metabolism of xenobiotics by cytochrome P450, and chemical carcinogenesis.

## Data Availability

The datasets presented in this study can be found in online repositories. The names of the repository/repositories and accession number(s) can be found in the article/supplementary material.

## References

[B1] Barrett T. Wilhite S. E. Ledoux P. Evangelista C. Kim I. F. Tomashevsky M. (2013). NCBI GEO: archive for functional genomics data sets--update. Nucleic Acids Res. 41, 991–995. 10.1093/nar/gks1193 PMC3531084 23193258

[B2] Brito C. Y. Melian C. Wagner A. M. (2016). Study of the pathogenesis and treatment of diabetes mellitus through animal models. Endocrinol. Nutr. 63, 345–353. 10.1016/j.endonu.2016.03.011 27246633

[B3] Chauche C. Nogales A. Zhu H. Goldfarb D. Ahmad S. A. I. Gu Q.(2017). Mammalian adaptation of an avian influenza A virus involves stepwise changes in NS1. J. Virol. 92,018755-e1917. 10.1128/JVI.01875-17 PMC5809720 29237841

[B4] Chen W. Tian T. Wang S. Xue Y. Sun Z.(2017). Characteristics of carotid atherosclerosis in elderly patients with type 2 diabetes at different disease course, and the intervention by statins in very elderly patients patients with type 2 diabetes at different disease course, and the intervention by statins in very elderly patients. J. Diabetes Investig. 9,389–395. 10.1111/jdi.12710 PMC5835477 28685957

[B5] Cheng X. Yi L. L. Zhuo Y. C. Bo X. Zhao Z. Li A. (2022). Network pharmacology and molecular docking to elucidate the potential mechanism of ligusticum chuanxiong against osteoarthritis. Front. Pharmacol. 13, 854215. 10.3389/fphar.2022.854215 35496280 PMC9050356

[B6] Da F. A. C. P. Mastronardi C. Johar A. Arcos B. M. Paz F. G.( 2017). Genetics of non-syndromic childhood obesity and the use of high-throughput DNA sequencing technologies childhood obesity and the use of high-throughput DNA sequencing technologies. J. Diabetes Complicat. 31,1549–1561. 10.1016/j.jdiacomp.2017.04.026 28735903

[B7] Dale T. Clarke P. A. Esdar C. Waalboer D. Adeniji P. O. Ortiz R. M. J. (2015).A selective chemical probe for exploring the role of CDK8 and CDK19 in human disease. Nat. Chem. Biol.11,973–980. 10.1038/nchembio.1952 26502155 PMC4677459

[B8] Doniz P. L. Martinez J. V. Nino M. P. Abud M. C. Hernandez C. B. Gonzalez A. R. (2011). Expression and function of Cbl-b in T cells from patients with systemic lupus erythematosus, and detection of the 2126 A/G Cblb gene polymorphism in the Mexican mestizo population erythematosus, and detection of the 2126 A/G Cblb gene polymorphism in the Mexican mestizo population. Lupus. 20,628–635. 10.1177/0961203310394896 21558139

[B9] Guess N. D. (2018). Dietary interventions for the prevention of type 2 diabetes in high-risk groups: current state of evidence and future research needs. Nutrients 10, 1245. 10.3390/nu10091245 30200572 PMC6163866

[B10] Halupa A. Bailey M. L. Huang K. Iscove N. N. Levy D. E. Barber D. L.(2005). A novel role for STAT1 in regulating murine erythropoiesis: deletion of STAT1 results in overall reduction of erythroid progenitors and alters their distribution progenitors and alters their distribution. Blood. 105,552–561. 10.1182/blood-2003-09-3237 15213094

[B11] Hiroki M. Kazuhiro K. (2022). Diversity of pathophysiology in type 2 diabetes shown by islet pathology. J. Diabetes Investig. 13, 6–13. 10.1111/jdi.13679 PMC8756316 34562302

[B12] Huang D. W. Sherman B. T. Tan Q. Collins J. R. Alvord W. G. Roayaei J. (2007). The DAVID Gene Functional Classification Tool: a novel biological module-centric algorithm to functionally analyze large gene lists. Genome Biol. 8, R183. 10.1186/gb-2007-8-9-r183 17784955 PMC2375021

[B13] Jae H. P. Woo Y. P. Hyun W. P.(2020). Cancer metabolism: phenotype, signaling and TherapeuticTargets. Cells 9, 2308. 10.3390/cells9102308 33081387 PMC7602974

[B14] Joanne B. C. Jose C. F. (2020). Genetics of diabetes mellitus and diabetes complications. Nat. Rev. Nephrol. 16, 377–390. 10.1038/s41581-020-0278-5 32398868 PMC9639302

[B16] Kaul N. Ali S. (2015). Genes, genetics, and environment in type 2 diabetes: implication in personalized medicine. DNA Cell Biol. 35, 1–12. 10.1089/dna.2015.2883 26495765

[B17] Le M. B. Vidal H. Naville D.(2018). Environmental pollutants and metabolic disorders: the multi-exposure scenario of life multi-exposure scenario of life. Front. Endocrinol. (Lausanne).9,582. 10.3389/fendo.2018.00582 30333793 PMC6176085

[B18] Li H. (2012). Systems genetics in "-omics" era: current and future development. Theory Biosci. 132, 1–16. 10.1007/s12064-012-0168-x 23138757

[B19] Linan R. L. Hernandez C. B. Doniz P. L. Portillo S. H. Baranda L. Cruz M. M. E. (2015). Analysis of expression and function of the co-stimulatory receptor SLAMF1 in immune cells from patients with systemic lupus erythematosus (SLE). Lupus. 24, 1184–1190. 10.1177/0961203315584412 25920347

[B20] Olferiev M. Masuda E. Tanaka S. Blank M. C. Pricop L. (2007). The role of activating protein 1 in the transcriptional regulation of the human FCGR2B promoter mediated by the -343 G -> C polymorphism associated with systemic lupus erythematosus. J. Biol. Chem. 282, 1738–1746. 10.1074/jbc.M605808200 17130130

[B21] Ridker P. M. Pare G. Parker A. Zee R. Y. Danik J. S. Buring J. E. (2008). Loci related to metabolic-syndrome pathways including LEPR,HNF1A, IL6R, and GCKR associate with plasma C-reactive protein: the Women's Genome Health Study. Am. J. Hum. Genet. 82,1185–1192. 10.1016/j.ajhg.2008.03.015 18439548 PMC2427311

[B22] Sanae E. B. Matthew L. S. Paulo S. P.(2019). Role of pre-existing type 2 diabetes in colorectal cancer survival among older Americans: a SEER-Medicare population-based study 2002-2011 survival among older Americans: a SEER-Medicare population-based study 2002-2011. Int. J. Colorectal Dis.34,1467–1475. 10.1007/s00384-019-03345-8 31289849

[B23] Shannon P. Markie A. Ozier O. Baliga N. S. Wang J. T. Ramage D. (2003). Cytoscape: a software environment for integrated models of biomolecular interaction networks. Genome Res. 13 (11), 2498–2504. 10.1101/gr.1239303 14597658 PMC403769

[B24] Shen H. Zhao J. Liu Y. Sun G.(2018).Interactions between and shared molecular mechanisms of diabetic peripheral neuropathy and obstructive sleep apnea in type 2 diabetes patients diabetic peripheral neuropathy and obstructive sleep apnea in type 2 diabetes patients. J. Diabetes Res. 2018,3458615. 10.1155/2018/3458615 30116739 PMC6079583

[B25] Stephanou A. Latchman D. S. Isenberg D. A. (1998). The regulation of heat shock proteins and their role in systemic lupus erythematosus. Semin. Arthritis Rheum. 28, 155–162. 10.1016/s0049-0172(98)80032-2 9872476

[B26] Vyas R. Bapat S. Jain E. Tambe S. S. Karthikeyan M. Kulkarni B. D. (2015). A study of applications of machine learning based classification methods for virtual screening of lead molecules. Comb. Chem. High. Throughput Screen 18, 658–672. 10.2174/1386207318666150703112447 26138573

[B27] Wang C. H. Wei Y. H.(2017). Role of mitochondrial dysfunction and dysregulation of Ca2+ homeostasis in the pathophysiology of insulin resistance and type 2 diabetes homeostasis in the pathophysiology of insulin resistance and type 2 diabetes. J. Biomed. Sci.24:70. 10.1186/s12929-017-0375-3 28882140 PMC5588717

[B28] Wang J. Shannon M. F. Young I. G. (2006). A role for Ets1, synergizing with AP-1 and GATA-3 in the regulation of IL-5 transcription in mouse Th2 lymphocytes regulation of IL-5 transcription in mouse Th2 lymphocytes. Int. Immunol. 18,313–323. 10.1093/intimm/dxh370 16373364

[B29] Xu G. Ni Z. Shi Y. Sun X. Wang H. Wei C. (2014). Screening essential genes of *Mycobacterium tuberculosis* with the pathway enrichment method. Mol. Biol. Rep. 41, 7639–7644. 10.1007/s11033-014-3654-z 25098602

[B30] Yasuda M. Tanaka Y. Ryu M. Tsuda S. Nakazawa T. (2014). RNA sequence reveals mouse retinal transcriptome changes early after axonal injury transcriptome changes early after axonal injury. PLoS One 9, e93258. 10.1371/journal.pone.0093258 24676137 PMC3968129

[B31] Yi F. H. Ni Z. Yao Q. G. Dan W. Xiao X. Q. Wen S. Z. (2022). Identification of the shared gene signatures and biological mechanism in type 2 diabetes and pancreatic cancer. Front. Endocrinol. (Lausanne) 13, 847760. 10.3389/fendo.2022.847760 35432196 PMC9010232

[B32] Zhang K. Han Y. Zhao Y. Sun Y. Zou M. Fu Y. (2019). Upregulated gga-miR-16-5p inhibits the proliferation cycle and promotes the apoptosis of MG-infected DF-1 cells by repressing PIK3R1-mediated the PI3K/akt/NF-κB pathway to exert anti-inflammatory effect. Int. J. Mol. Sci. 20, 1036. 10.3390/ijms20051036 30818821 PMC6429190

[B33] Zhao F. Xuan Z. Liu L. Zhang M. Q. (2005). TRED: a Transcriptional Regulatory Element Database and a platform for *in silico* gene regulation studies. Nucleic Acids Res. 33 (Databaseissue), D103–D107. 10.1093/nar/gki004 15608156 PMC539958

[B34] Zhao M. Wang H. Chen J. Xi Y. Wang F. Huo C. (2021). Expression of long non-coding RNA H19 in colorectal cancer patients with type 2 diabetes. Arch. Physiol. Biochem. 127, 1–7. 10.1080/13813455.2019.1628068 31232113

